# Bioaccessibility and Human Exposure Assessment of Cadmium and Arsenic in Pakchoi Genotypes Grown in Co-Contaminated Soils

**DOI:** 10.3390/ijerph14090977

**Published:** 2017-08-29

**Authors:** Yanyan Wei, Xiaoman Zheng, Md. Jahidul Islam Shohag, Minghua Gu

**Affiliations:** 1 State Key Laboratory for Conservation and Utilization of Subtropical Agro-Bioresources, Cultivation Base of Guangxi Key Laboratory for Agro-Environment and Agro-Products Safety, College of Agriculture, Guangxi University, Nanning 530004, China; yanyanwei@gxu.edu.cn (Y.W.); xiaoman_zheng@126.com (X.Z.); 2 Ministry of Education Key Laboratory of Environmental Remediation and Ecosystem Health, College of Environmental and Resources Science, Zhejiang University, Hangzhou 310058, China; jshohag@gmail.com; 3 Department of Agriculture, Bangabandhu Sheikh Mujibur Rahman Science and Technology University, Gopalganj 8100, Bangladesh

**Keywords:** bioaccessibility, cadmium, arsenic, pakchoi, genotype, human exposure

## Abstract

In many countries cadmium (Cd) and arsenic (As) commonly coexist in soils contaminated by mining activities, and can easily enter the human body via consumption of leafy vegetables, like the popularly consumed pakchoi (*Brassica chinensis* L.), causing major health concerns. In the present study, bioaccessibility and human exposure of Cd and As were assessed in twenty genotypes of pakchoi cultured at two different levels of co-contamination to identify low health risk genotypes. The bioaccessibilities of Cd and As represent a fraction of the total metals content could be bioaccessible for human, in the present study, significant differences in pakchoi Cd and As bioaccessibility were observed among all tested genotypes and co-contaminated levels. Cd and As bioaccessibility of pakchoi were in the ranges of 24.0–87.6% and 20.1–82.5%, respectively, for in the high level co-contaminated soils, which was significantly higher than for low level co-contaminated soils with 7.9–71.8% for Cd bioaccessibility and 16.1–59.0% for As bioaccessibility. The values of bioaccessible established daily intakes (BEDI) and the total bioaccessible target hazard quotients (TBTHQ) of Cd and As were also considerably higher in high level co-contaminated soils than in low level co-contaminated soils. Two genotypes (Meiguanqinggengcai and Zhenqing60F1) contained relatively low concentrations and bioaccessible Cd and As and, their BEDI and TBTHQ for Cd and As ranged below the tolerable limits set by the FAO/WHO (BEDI of Cd < 0.83 μg kg^−1^ bw day^−1^, BEDI of As < 3 μg kg^−1^ bw day^−1^) and United States Environmental Protection Agency (TBTHQ for Cd and As < 1), this applied for both levels of co-contaminated soils for adults and children. Consequently, these findings suggest identification of safe genotypes in leafy vegetable with low health risk via genotypic screening and breeding methods could be a useful strategy to ensure the safety of food crops grown in those Cd and As co-contaminated fields due to mining activities.

## 1. Introduction

Contamination of agricultural soils by heavy metal(loid)s is a significant global environmental problem. Cadmium (Cd) and arsenic (As) are the most closely monitored metals, and commonly coexist in the contaminated soils in many countries, due to large-scale mining and metal processing activities, thus, the coexistence of Cd and As in mine-impacted areas has received considerable attention all around in the world. In Spain, agricultural soils surrounding mines have been reported as polluted with As (77–2649 mg kg^−1^) and Cd (3–17 mg kg^−1^) [1]. About 1000 abandoned metal mines exist in South Korea, where the levels of As and Cd reached up to 626 and 1.4 mg·kg^−1^ in the soil surrounding the these mines [2]. In northern Vietnam, the content of As and Cd in mining-impacted farmland area ranged between 16.3–1010 mg kg^−1^ and 0.55–3.25 mg kg^−1^ [3]. In China, survey results have shown that there are approximately 14,000,000 ha of sites contaminated by As, Cd and other heavy metals, involving 25 areas in 11 provinces [4], especially in southern China. Li et al. [5] investigated 71 mine areas in China and found that the means of As and Cd in soils surrounding these mines, reached up to 195.5 and 11 mg·kg^−1^, respectively, which considerably exceeded the farmland environmental quality evaluation standards for edible agricultural products in China (the critical Cd level in soils is as low as <0.30 mg kg^−1^, the critical As level in soils is as low as <30 mg kg^−1^) [6]. In China, Guangxi province is considered as the most heavily As and Cd polluted province [5]. There are currently over 6800 mines in operation in Guangxi province, which ranks first in China in terms of As, Mn and Sn mining, destroying a total land area of about 600,000 ha [7].

Heavy metals in mine-impacted areas may enter to the human body through consumption of food crops, such as vegetables. Exceedance of the contaminant limits in vegetables is widespread in mine-impacted areas, especially in southern China, due to the elevated inputs of contaminants caused by mining activities, and the acidic nature of the soil [8,9]. In mining impacted areas of Guangxi, Yu et al. [10] reported that 7.1% and 35.7% of leafy vegetable samples exceeded the maximum levels for food contaminants in China for As and Cd (allowing a Cd concentration in vegetables <0.2 mg kg^−1^, As concentration in vegetables <0.5 mg kg^−1^) [11]. Prolonged consumption of contaminated food crops may lead to human health problems, and clinical studies have associated Cd and As with various forms of cancers, nephrotoxicity, hepatotoxicity, neurologic and neurobehavioural disorders, as well as cardiovascular diseases in humans [12]. Thus, particular attention should be focused on vegetable safety in mine-impacted areas. Pakchoi (*Brassica chinensis* L.) is an important leafy vegetable consumed around the world, being among the 10 most important consumed vegetables in East, Northeast and Southeast countries, and having been introduced successfully in Europe, America and Australia, is increasing in prominence in Western diets [13]. Previous studies reported that among vegetable species, pakchoi is more vulnerable to Cd and As in contaminated soils [14,15]. Thus, developing of technologies that lower the risk of Cd and As through the food chain, and pakchoi in particular, is an urgent task.

Numerous techniques have been developed in this regard, such as phytoremediation and phytoextraction [16]. However, these techniques are difficult to implement in contaminated farmland of developing countries due to their high costs and long duration. Screening and breeding of vegetable genotypes with low Cd and As pollution risk offers a low-cost strategy that could impede the transfer of these metals into the food chain [8,17]. In Nature, soils are contaminated by several heavy metals, some of which are highly toxic. However until now, most of the conducted studies mainly focused on single toxic metals such as As or Cd, rather than investigating a combination of both. In addition, pakchoi consumption could contribute highly to the estimated daily intake of Cd and As, which poses potential risk associated with human health [18,19]. However, little is known about the effects of different genotypes on the bioaccessibility of Cd and As as well as their health risks via consumption of pakchoi cultured on Cd and As co-contaminated soils.

Many methods have been developed to estimate the potential health risks of the consumption of contaminated vegetables. In general, the total amount of heavy metals in the food is commonly used as a measure of heavy metal contamination in a food matrix. In fact, the health risks of heavy metal consumption depend on the fraction of a compound released from its matrix in the gastrointestinal tract making it available for absorption, and only a fraction of the total metal content will be bioaccessible for humans [20,21]. Bioaccessibility in turn is an estimation of maximum oral bioavailability of the contaminant in food and can therefore be used as an accurate tool to assess health risks [22,23].

In vitro digestion methods, based on the human gastrointestinal system, have been proposed and successfully used to study the bioaccessibility and risk assessment of contaminants (e.g., metals) from various soils and foods [20,21,22,23,24,25]. With regard to heavy metals in food, a limited number of Cd and As bioaccessibility studies have been reported, so far, mostly concerning rice [22,26,27], mushrooms [28], edible seaweeds [29,30], and seafood [31]. Only few studies have investigated the bioaccessibility of Cd and As in vegetables [23,32,33], and as previously mentioned, most of these studies only focused on the bioaccessibility of single toxic metals. This highlights the importance for more bioaccessibility studies of the combined effects of Cd and As in pakchoi to improve the risk assessment.

Consequently, the main objectives of the present study were: (i) to measure biomass production and bioaccessibility of Cd and As in twenty genotypes of pakchoi cultured on two different levels of co-contaminated soils from mine-impacted areas based on in vitro digestion; (ii) to assess human health risks based on the Cd and As bioaccessibility via consumption of these pakchoi genotypes, identifying the Cd + As safer genotypes. Results from this study provide insight into possibilities for using genotypic screening and breeding methods in pakchoi, as well as other leafy vegetables, with low health risk to ensure food safety in Cd and As co-contaminated regions due to mining activities in the world.

## 2. Materials and Methods

### 2.1. Plant Material

Seeds of twenty pakchoi genotypes (Table 1) were collected from seed markets of China. All selected genotypes were widely used by farmers.

### 2.2. Greenhouse Experiment

Two levels of soils co-contaminated with Cd and As used for cultivation were collected from the top layer (0–20 cm) of vegetable fields located in areas with high mining activities in Nandan County of Guangxi province. The county is located in the south of China, renowned as a metal-rich-area, where is densely polluted by Cd and As as previously reported [34]. All of these selected soil types were of acidic nature, which is the main soil type distributed in many mining areas of southern China. Soil samples were air dried, ground and passed through 10, 80, or 100 mesh stainless steel sieves in accordance with different analysis procedures. Basic soil characteristics of representative samples were then measured according to the procedures described by Liu et al. [15] and Khan et al. [35]. Soil samples were defined as low level co-contaminated soil (L-C soil), that had a total Cd concentration of 0.61 mg kg^−1^, a total As concentration of 35.0 mg kg^−1^, a soil pH of 6.3, organic matter of 1.4 g kg^−1^, total N of 1.6 g kg^−1^, and total P of 3.1 g kg^−1^. Soil samples that were defined as high level co-contaminated soil (H-C soil) had a total Cd concentration of 2.39 mg kg^−1^, a total As concentration of 82.5 mg kg^−1^, a soil pH 6.5, organic matter of 2.1 g kg^−1^, total N of 1.1 g kg^−1^, and total P of 5.4 g kg^−1^. The L-C and H-C soils exceeded the maximum levels allowed by the farmland environmental quality evaluation standards for edible agricultural products of China [6].

The co-contaminated soil were air dried and passed through a 2-mm sieve for a greenhouse experiment. A pot experiment was conducted, culturing 20 pakchoi genotypes in plastic pots from September to November 2016 at Guangxi University (Nanning, China). For each pakchoi genotype, about 20 seeds were pre-soaked in water for 3 h at room temperature and then sown into each plastic pot previously filled with 8.0 kg of soil co-contaminated with Cd and As. Basal fertilizers of 0.24 g kg^−1^ N as urea, 0.12 g kg^−1^ P_2_O_5_ as KH_2_PO_4_ and 0.24 g kg^−1^ K_2_O as KH_2_PO_4_ and KCl were placed into each plastic pot before planting. The pot experiment was conducted with three replicates positioned randomly in a greenhouse under controlled conditions of 16 h of light at 30 °C and 8 h of dark at 22 °C, and the plants were regularly monitored and daily irrigated with water.

### 2.3. Sample Preparation

After 60 days, the edible parts of these 20 pakchoi genotypes were harvested and washed with deionized water. Each sample was homogenized and divided into two portions. One portion of the sample was digested to determine Cd and As concentrations, the other portion of the same sample was stored at 4 °C for further evaluation of Cd and As bioaccessibility.

### 2.4. Determination of Cd and As Concentrations

Pakchoi samples (0.3 g) were predigested overnight in nitric acid (5 mL) in microwave digestion tubes at room temperature. Then the solution was heated (to 180 °C) in a microwave oven (MARS 6 240/50, CEM Corporation, Matthews, NC, USA). Triplicate analyses were performed for each sample for quality control. The Cd and As concentrations of pakchoi samples were analyzed via an inductively-coupled plasma mass spectrometer (ICP-MS, NEXION 350X, PerkinElmer Life Science Incorporated, Waltham, MA, USA). Blank and drift standards were run after three determinations, respectively to calibrate the instrument. Reagent blanks and the standard reference vegetable samples GBW10048 (GSB-26) and GBW10014 (GSB-5) were also included in each batch. The recovery rates of Cd and As in standard reference vegetable samples were in ranged between 98.12–109% and 92.56–97.34%, respectively.

### 2.5. In Vitro Evaluation of Bioaccessibility

The bioaccessibility of Cd and As in the vegetable samples was determined via the physiologically based extraction test (PBET) method, following previously described protocols [24,32,36]. Overall, PBET mimics the human gastrointestinal tract, which consists of two phases including the gastric and gastrointestinal phases. Briefly, the prepared pakchoi samples were subjected to simulated gastric digestion by incubating them in 25 mL of gastric solution. The gastric solution contained 1.25 g L^−1^ of pepsin, 0.50 g L^−1^ of citric acid, 0.50 g L^−1^ of malic acid, 420 μL L^−1^ of dl-lactic acid and 500 μL L^−1^ of acetic acid dissolved in deionized water. The pH was adjusted to 1.5 with 6 M HCl. The mixture was incubated in a shaking water bath at 37 °C at 100 rpm for 2 h. Then the solution was centrifuged at 4000 rpm for 10 min and an aliquot (5 mL) was collected from the solution and filtered through a 0.45 μm filter disk for further analysis. At the gastrointestinal stage, freshly prepared intestinal solution (2.5 g salt bile extract and 0.75 g pancreatin dissolved in 25 mL of 0.1 mol L^−1^ NaHCO_3_) was added and the pH of the mixture was raised to pH 7 via dropwise addition of 0.5 M NaOH. Samples were incubated in a shaking water bath at 200 rpm for 4 h at 37 °C. Aliquots (5.0 mL) of the sample solutions were collected and filtered through (0.45 μm). The extracts were kept at 4 °C prior to analysis of Cd and As concentrations via ICP-MS. The bioaccessibilities (%) of Cd and As in each pakchoi sample were calculated as a percentage and calculated per digestion using the following equation [32]:

$$\mathrm{Bioaccessibility}(\%)=\frac{\mathrm{Bioaccessible}\;\mathrm{metal}\;\mathrm{concentration}}{\mathrm{Total}\;\mathrm{metal}\;\mathrm{concentration}\;\mathrm{in}\;\mathrm{vegetables}}\times 100\%$$

### 2.6. Health Risk Assessment

To evaluate potential human exposure to Cd or As via human consumption of the resulting pakchoi samples by the consumers, the bioaccessible established daily intake (BEDI) for Cd and As [26], based on the bioaccessibility data, were calculated via using the following equation:

$$\mathrm{BEDI}=\frac{\mathrm{RC}\times \mathrm{BC}}{\mathrm{BW}}$$

where BC represents the bioaccessible concentrations of metals in ingestion (mg kg^−1^), RC represents the daily vegetable consumption (g person^−1^ d^−1^), BW is average body weight (60 kg for adults and 32.5 kg for children). Vegetable consumptions of 394 g d^−1^ for adults and 257 g d^−1^ for children assumed, following Song et al. [37].

To evaluate a long-term and co-risk potential hazardous exposure to Cd and As via consumption of vegetable by the consumers, the bioaccessible target hazard quotient (BTHQ) and the total BTHQ (TBTHQ) [32,38] were calculated based on the bioaccessibility data, using the following equations:

$$\mathrm{BTHQ}=\frac{\mathrm{ED}\times \mathrm{EF}\times \mathrm{BEDI}}{\mathrm{RfD}\times \mathrm{AT}\times 10^{-3}}$$

$$\mathrm{TBTHQ}=\mathrm{BTH}Q_{\mathrm{Cd}}+\mathrm{BTH}Q_{\mathrm{As}}$$

where ED represents the exposure duration (70 years), EF represents the exposure frequency (365 days year^−1^), AT represents the average time for noncarcinogens (365 days year^−1^ × exposure duration), 10^−3^ represents the unit conversion factor, and RfD represents corresponding oral reference dose (1 and 0.3 μg kg^−1^ day^−1^ for Cd and As, respectively), as suggested by the United States Environmental Protection Agency (USEPA) [39].

### 2.7. Statistical Analysis

Statistical analysis of the data was performed via SPSS 12.0 (SPSS, Inc., Chicago, IL, USA). All data were subjected to a separate analysis of variance (ANOVA) for each genotype, and Fisher’s least significant difference (LSD) at *p* < 0.05 was used to determine the differences between treatment means.

## 3. Results and Discussion

### 3.1. Shoot Biomass

**Figure 1:**
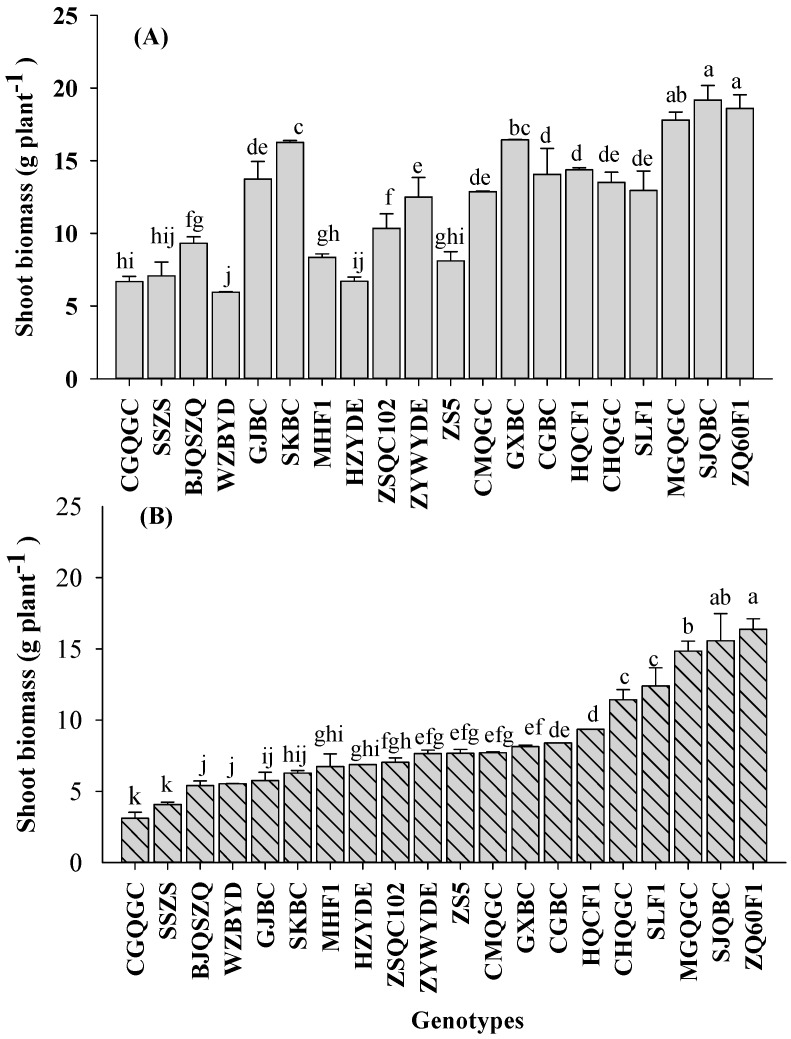
Shoot biomasses of 20 pakchoi genotypes grown at low- (**A**) and high- (**B**) levels of Cd and As co-contaminated soils. Error bars depict standard errors of the means (*n* = 3). Different letters indicate significant difference among genotypes according to LSD test (*p* < 0.05).

Significant differences (*p* < 0.05) in biomass were observed among all 20 pakchoi genotypes, which were grown in both Cd and As co-contaminated soils (Table 2, Figure 1). Under the L-C soil conditions, shoot biomasses of pakchoi ranged from 5.6 to 19.2 g plant^−1^ among all 20 genotypes, while under H-C soil conditions, shoot biomasses of pakchoi ranged from 3.1 to 16.36 g plant^−1^ among all 20 genotypes. Twelve genotypes yielded significantly lower shoot biomasses under the H-C soil condition than those under the L-C soil condition (*p* < 0.05), however, shoot biomasses of eight genotypes did not decrease significantly under H-C soil treatment, compared to that in L-C soil. Three genotypes, namely Sijiqingbaicai, Meiguanqinggengcai and Zhenqing60F1, had relatively high yield under both levels of Cd and As co-contaminated soils. The result agreed with results of previous studies [17,19,40], suggesting that heavy metal co-contamination in soil, may not be detectable by simply assessing the plant appearance, and food safety could continue to be threatened by the consumption of contaminated pakchoi.

### 3.2. Concentrations of Cd and As in Pakchoi Shoots

**Figure 2:**
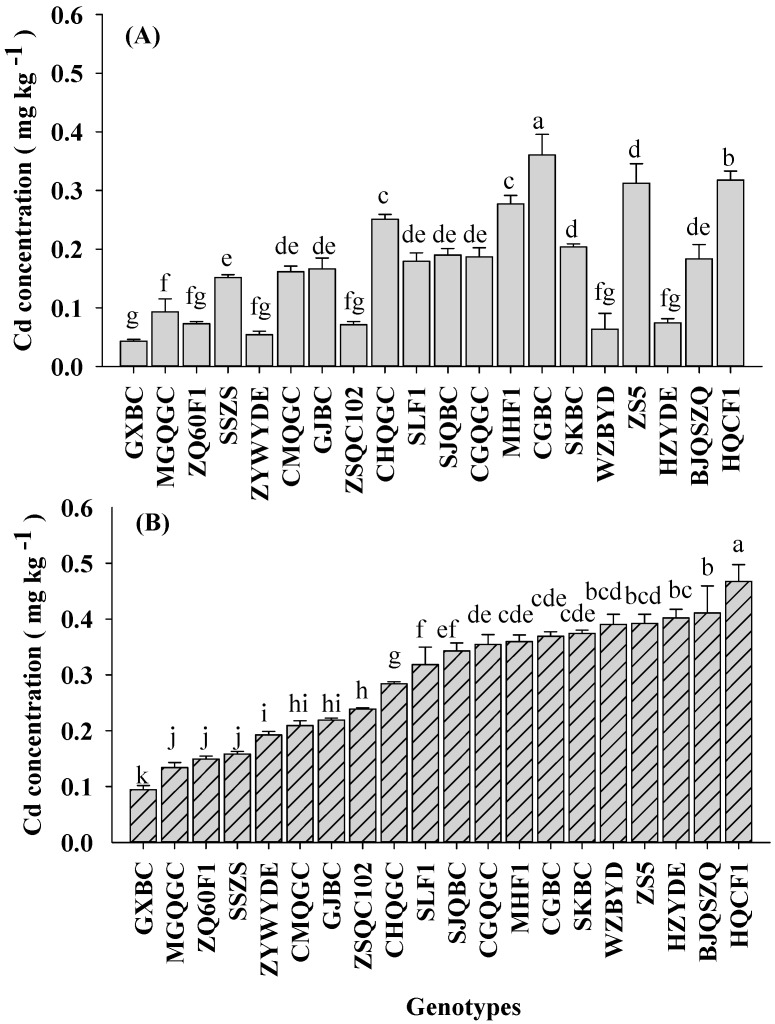
Shoot Cd concentrations in pakchoi among 20 genotypes grown in low-level co-contaminated soil (**A**), and high-level co-contaminated soil (**B**). Error bars depict standard errors of the means (*n* = 3). Different letters depict significant difference among genotypes according to LSD test (*p* < 0.05).

**Figure 3:**
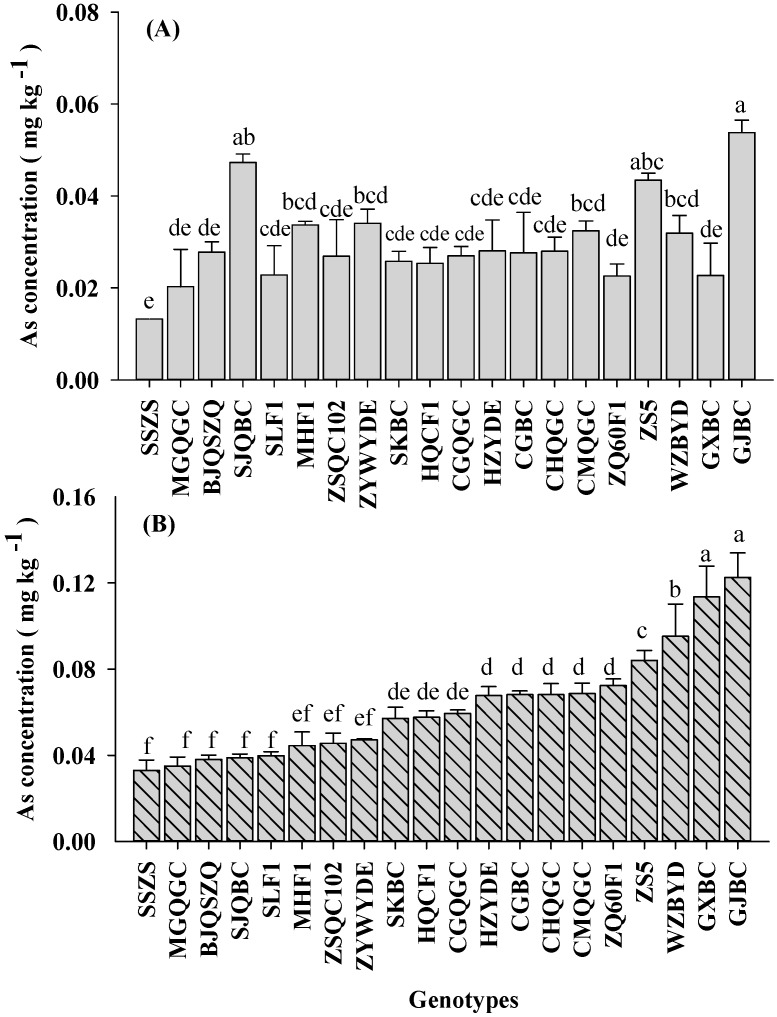
Shoot As concentrations in pakchoi among 20 genotypes grown in low-level co-contaminated soil (**A**) and high-level co-contaminated soil (**B**). Error bars depict standard errors of the means (*n* = 3). Different letters depict significant difference among genotypes according to the LSD test (*p* < 0.05).

Soil treatment with different Cd and As levels significantly affected Cd and As accumulation in pakchoi shoots (Table 2, Figure 2 and Figure 3). The average shoot Cd concentrations (FW basis) in L-C soil (0.17 mg kg^−1^) were lower than those for H-C soil (0.29 mg kg^−1^). A similar trend was found in case of As concentration of pakchoi with the increasing total As levels in the soil. The average shoot As concentrations were 0.03 mg kg^−1^ under L-C soil, and 0.06 mg kg^−1^ under H-C soil. These results were consistent with the previous reports [14,15].

Significant genotypic variation (*p* < 0.05) in Cd and As concentrations in the edible parts of pakchoi were observed (Table 2, Figure 2 and Figure 3). Eight-fold (0.04–0.36 mg kg^−1^) and 5-fold (0.09 to 0.47 mg kg^−1^) variations of shoot Cd concentrations among all 20 sampled pakchoi genotypes were observed in L-C soil and H-C soil, respectively. According to the published maximum levels of contaminants in foods of China [11], Cd concentration in vegetables should be below 0.2 mg kg^−1^ FW. However, the present data showed that Cd concentration in 75% of the pakchoi genotypes grown in H-C soil were exceeded this threshold, higher than those of L-C soil (30%). These results agreed with previous studies, reporting that leafy vegetables, such as pakchoi, could easily uptake Cd from soils [41,42,43].

Furthermore, As accumulation was not as excessive as Cd accumulation in pakchoi under these co-contaminated soil conditions (Figure 3), as concentrations in pakchoi reached up to 0.05 mg kg^−1^ under the L-C soil condition, and 0.12 mg kg^−1^ under the H-C soil condition. Four-fold variations of As concentration were observed among the genotypes grown in both levels of co-contaminated soils. As concentrations in all of these selected pakchoi genotypes were within the threshold (<0.5 mg kg^−1^ FW) [11]. Evidently, the response of different pakchoi genotypes was more sensitive to Cd concentrations than to As concentrations. Similar studies reported that some crop genotypes have different abilities to uptake and accumulate of Cd [17,40,43], and As [44,45]. The results of this study suggested that pakchoi could simultaneously accumulate high concentrations of Cd and As. Thus, pakchoi grown on such Cd and As co-contaminated soils around the mine could be potential contributors to dietary Cd and As exposure in the population, therefore, human risk of Cd and As co-contamination via consumption of these pakchoi genotypes should be considered.

### 3.3. Bioaccessibility of Cd and As in 20 Pakchoi Genotypes

**Figure 4:**
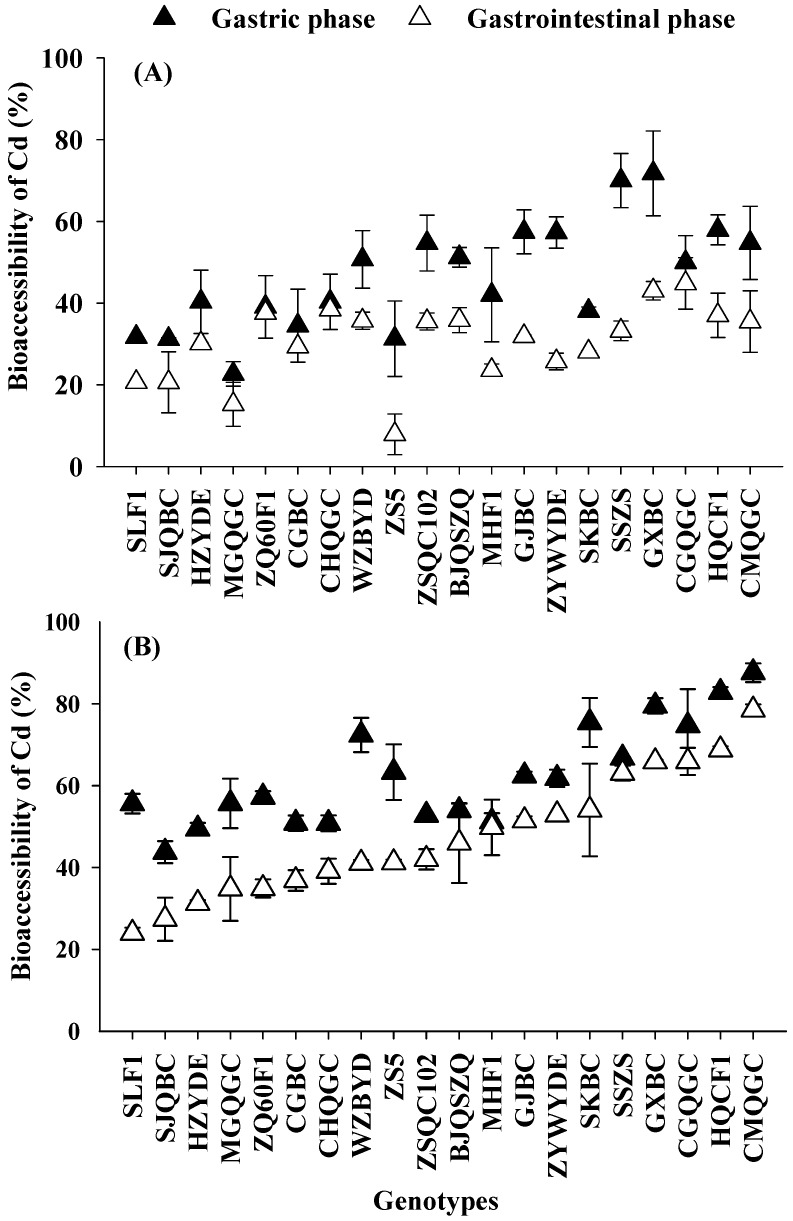
Cd bioaccessibility during gastric phase and gastrointestinal phase of pakchoi of 20 genotypes, grown in low-level co-contaminated soil (**A**) and high-level co-contaminated soil (**B**). Error bars depict standard errors of the means (*n* = 3).

**Figure 5:**
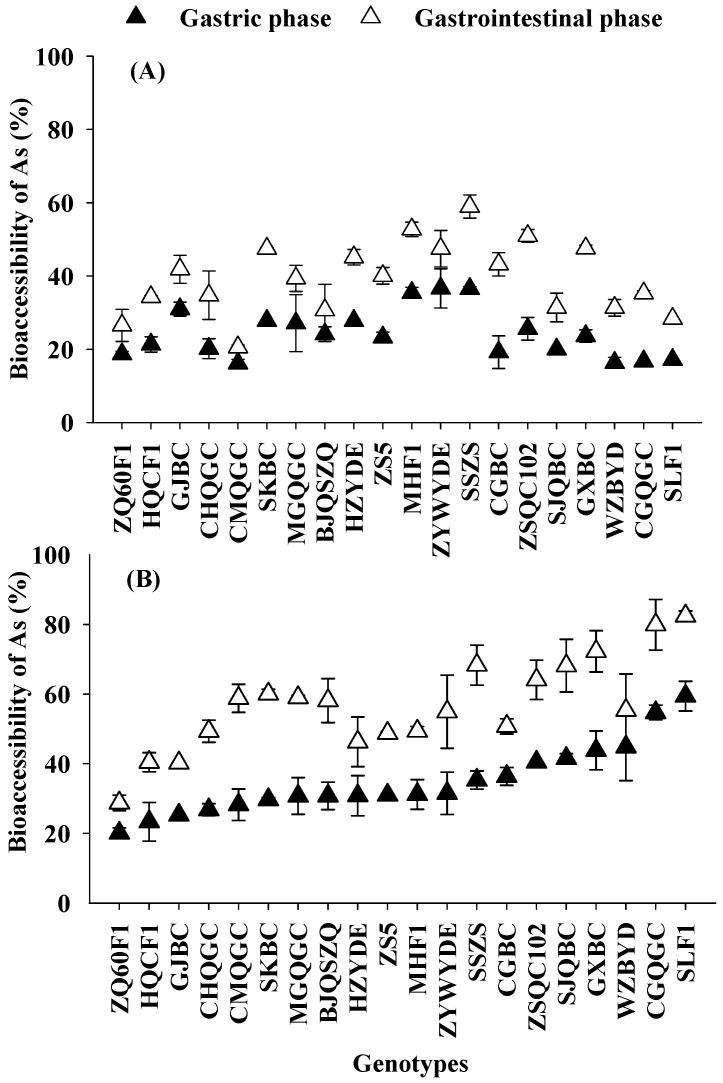
Arsenic bioaccessibility (%) during gastric phase and gastrointestinal phase of pakchoi among 20 genotypes grown on at low-level co-contaminated soil (**A**) and high-level co-contaminated soil (**B**). Error bars depict standard errors of the means (*n* = 3).

The percentages of Cd and As bioaccessibility in pakchoi among 20 genotypes measured in the gastric and gastrointestinal phases were presented in Figure 4 and Figure 5.

High percentages of Cd bioaccessibility were observed during in vitro digestion, the average Cd bioaccessibility of the pakchoi was higher during gastric phase (54.37%) than during the gastrointestinal phase (38.97%) (Figure 4). These observations were similar to previous reports [22,23,33,46]. The results may be related to the fact that most of the Cd was accumulated in the cell vacuoles of the plants, except for a subset, which is absorbed by the cell wall; hence, amounts of Cd are easily released from plant tissues during in vitro digestion [46,47]. During the gastric phase, enzymes (e.g., pepsin) and low pH were able to release most of the Cd and only a small portion still remained absorbed within plant tissues [26,33]. However, during gastrointestinal phase, increase in pH (from 1.5 in the gastric phase to 7.0 in the gastrointestinal phase) as well as addition of pancreatin and bile extracts might bring precipitation and/or resorption of part of the solubilized Cd, thus reducing Cd bioaccessibility during this phase [46,48].

Moreover, genotypes and soil treatment significantly affected Cd bioaccessibility in the gastric and gastrointestinal phases (*p* < 0.05) (Table 2, Figure 4). Under the L-C soil condition, the bioaccessibility of Cd in the 20 pakchoi genotypes varied between 22.7% and 71.8% during the gastric phase, and between 7.9% and 44.8% during the gastrointestinal phase. Under the H-C soil condition, the bioaccessibility of Cd varied between 43.8% and 87.6% during the gastric phase, and between 24.0% and 78.5% during the gastrointestinal phase. Under the L-C soil, Cd bioaccessibility varied 3-fold and 6-fold in gastric and gastrointestinal phases among all 20 genotypes. Under the H-C soil, Cd bioaccessibility varied 2-fold and 3-fold among twenty genotypes. In general, Cd bioaccessibility during gastric and gastrointestinal phases of pakchoi obtained from H-C soil were higher than those from L-C soil. Similar results have been reported for radish, where the variation in the concentrations of Cd in soils had the same impact on bioaccessibility [33].

Bioaccessibility in pakchoi varied from 16.1% to 59.03%, and 20.53% to 82.46% for gastric and gastrointestinal fractions, respectively. The values of As bioaccessibility in pakchoi obtained in the present research were in line with the previous reports for rice [26,27], mushroom [28] and seaweed [29]. Thus, a high variability in As bioavailability was observed depending on the different types of food studied. In contrast to Cd bioaccessibility, As bioaccessibility in the gastrointestinal phase was higher (up to 82.5%) than those during the gastric phase (up to 59.0%). An increased bioaccessibility value during the gastrointestinal phase has been noted for As in some types of foods [27,29]. It is likely that digestive enzymes from pancreas and bile in the gastrointestinal phase are involved in the breakdown of poly-saccharides into monosaccharides and in the cleavage of denaturalized proteins. Future release of the protein-bound fractions of As would consequently, increasing the As bioaccessibility during this phase [27]. Furthermore, significant differences (*p* < 0.05) were also found in As bioaccessibility among 20 genotypes grown in two levels co-contamination soils (Table 2, Figure 4). Two-fold and 3-fold genotypic variations in As bioaccessibility of pakchoi were obtained from L-C soil, in gastric and gastrointestinal phases, respectively. Under the H-C soil condition, 3-fold and 2-fold genotypic variations were observed in As bioaccessibility during gastric and gastrointestinal phases, respectively.

To date, limited data on the variation of Cd and As bioaccessibility in pakchoi genotypes that have been cultured in co-contaminated soil have been reported in the literature; therefore, the results obtained in this study cannot be compared to previously published standards. The present study found significant genotypic variations on Cd and As bioaccessible fractions, which was probably due to the differences in metal concentrations, metal species, the cell wall materials, and nutritional characteristics of pakchoi genotypes that might affect the metal bioaccessibility during digestion [33,49]. The present observations suggest that it is possible to select pakchoi genotypes (such as Meiguanqinggengcai and Zhenqing60F1) with relatively low levels of Cd and As bioaccessibility, even in the presence of a high levels of co-contaminated soil.

### 3.4. Health Risk Assessment of Cd and As in Pakchoi

**Figure 6:**
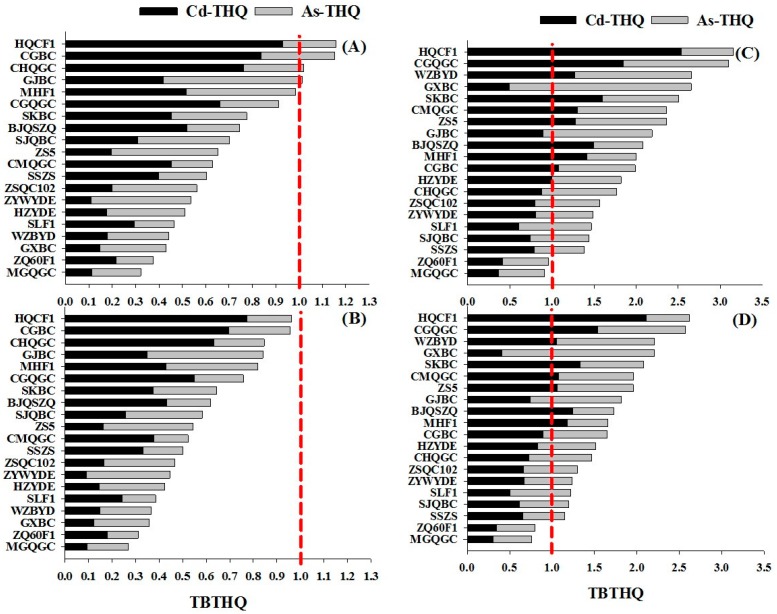
The total bioaccessible target hazard quotients (TBTHQs) of Cd and As via consumption of 20 pakchoi genotypes from low-level co-contaminated soils for children (**A**) and adults (**B**), as well as for high-level co-contaminated soil for children (**C**) and adults (**D**). The dotted red line represents for the tolerable limits of target hazard quotient (<1) set by USEPA [39].

The total Cd and As intake would lead to an overestimation of the absorbed amount since not all pollutants are bioaccessible. Based on the data of bioaccessibility, the bioaccessible established daily intakes (BEDI) and total bioaccessible target hazard quotients (TBTHQ) of Cd and As for local residents including adults and children via consumption of the tested pakchoi genotypes are presented in Table 3 and Figure 6. The Food and Agriculture Organization (FAO) and Word Health Organization (WHO) [50] has established a provisional tolerable weekly intake (PTWI) of 21 μg kg^−1^ body weight (bw) (equivalent to 3.0 μg kg^−1^ bw day^−1^, PTDI) for As and a provisional tolerable monthly intake (PTMI) of 25 μg kg^−1^ bw for Cd (equivalent to 0.83 μg kg^−1^ bw day^−1^, PTDI).

Generally, the BEDI values of Cd and As for adults and children via consumption of pakchoi that was cultured on L-C soil were lower than those on H-C soil (Table 3). In this L-C soil, the BEDI values of Cd for adults and children via pakchoi consumption varied between 0.09 and 0.77 μg kg^−1^ bw day^−1^, 0.11 and 0.93 μg kg^−1^ bw day^−1^, respectively. Meanwhile, the BEDI values of As were varied between 0.04 and 0.15 μg kg^−1^ bw day^−1^ for adults and, 0.05 and 0.18 μg kg^−1^ bw day^−1^ for children. All of the pakchoi genotypes cultured on L-C soil had lower BEDI values of As for adults and children than PTDI values, while 90% of pakchoi genotypes with BEDI values of Cd remained within safe PTDI value, The exception formed, the 2 genotypes HuoqingcaiF1 and Changgengbaicai, which had the high BEDI values of Cd (>0.83 μg kg^−1^ bw day^−1^, PTDI) for children. Additionally, under the H-C soil condition, the BEDI values of Cd via pakchoi consumption were varied from 0.31 to 2.11 μg kg^−1^ bw day^−1^ and, from 0.37 to 2.54 μg kg^−1^ bw day^−1^ for adults and children, respectively. BEDI values of Cd ranged from 50% to 60% of pakchoi genotypes for adults and children exceeded the PTDI value, while the BEDI values of As in all the tested the pakchoi genotypes remained below the PTDI value.

Total target hazard quotients are a reliable and helpful measure to assess combined risks of heavy metals from different foods [38]. A value of target hazard quotient below 1 indicates that the exposure population is unlikely to experience apparent adverse effects [38,39]. In the current study, the higher values of TBTHQ via consumption of pakchoi were observed in H-C soil compared to L-C soil (Figure 6). Under the L-C soil condition, the TBTHQ from most of the pakchoi genotype was below 1 for both adults and children, except for the four genotypes HuoqingcaiF1, Changgengbaicai, Gaojiaobaicai and Chunhuaqinggengcai. However, under the H-C soil condition, except for the two genotypes Meiguanqinggengcai and Zhenqing 60F1, 90% of genotypes had high TBTHQ values (>1), implying apparent adverse effects by co-exposure to Cd and As, even some of them with safe levels of BEDI of Cd and As. Accordingly, for the purpose of health protection against heavy metal toxicity, consumption levels of genotypes with high TBTHQ values (>1) need to be closely monitored.

The results for the BEDI and TBTHQ of Cd and As suggested that long-term large consumption of pakchoi originating from H-C soil imposes a higher risk than compared to those cultivated on L-C soil. Moreover, the BEDI and TBTHQ values of Cd and As were higher for children than for adults; thus, high exposure to these metals may raise some concerns about consumption by children. Under both levels of co-contaminated soils, two genotypes Meiguanqinggengcai and Zhenqing60F1, had BEDI values for Cd and As below the permissible limit set by the FAO/WHO for both adults and children, and their TBTHQ values were also below 1 for both adults and children, which identified them as safe genotypes, recommended for planting in co-contaminated regions.

## 4. Conclusions

High levels of potentially hazardous metals Cd and As are frequently observed in leafy vegetables grown in mining-impacted areas, such as the popularly consumed pakchoi. It’s important to minimizing the risk of Cd and As through consumption of pakchoi. The results of this study suggested that genotype dramatically affected oral bioaccessibility of Cd and As. Oral bioaccessibility of Cd and As of 20 pakchoi genotypes varied between 7.9% and 71.8% and, 16.1% and 59.0% in low level co-contaminated soil, and varied between 24.0% and 87.6%, 20.1% and 82.5% for high level co-contaminated soils, respectively. The BEDI and TBTHQ of Cd and As via pakchoi consumption suggested that pakchoi grown in these co-contaminated soils would not be safe for consumption, especially for the high levels of Cd and As co-contaminated soil condition. In this case, selecting appropriated genotypes according to the degree of heavy metal pollution in soils will benefit food security, while retaining relatively high yield. Two genotypes, Zhenqing60F1 and Meiguanqinggengcai grown under two levels of Cd and As co-contaminated soils, both contained relatively low concentrations and Cd and As with low bioaccessibility, and their BEDIs were all below the FAO/WHO permissible limit (BEDI of Cd < 0.83 μg kg^−^^1^ bw day^−^^1^, BEDI of As < 3 μg kg^−^^1^ bw day^−^^1^), moreover, their TBTHQs were below USEPA permissible limit (<1) for both adults and children. This identifies two pakchoi genotypes that could be used for safe pakchoi production and consumption in Cd and As co-contaminated regions (soil Cd concentration < 2.39 mg kg^−1^ and, soil As concentration < 82.5 mg kg^−1^). This finding highlights the identification of Cd+As safe genotypes in pakchoi, as well as other leafy vegetables, via genotypic screening and breeding methods can been utilized with low health risk to ensure food safety in areas with Cd and As co-contamination due to mining activities.

## Figures and Tables

**Table 1:** Pakchoi genotypes used in the experiments and their abbreviations.

Genotypes	Abbreviations	Genotypes	Abbreviations
Bujieqiusuzhouqing	BJQSZQ	MinhuangF1	MHF1
Changgengbaicai	CGBC	Shuikoubaicai	SKBC
Chunguanqinggengcai	CGQGC	Sijiqingbaicai	SJQBC
Chunhuaqinggengcai	CHQGC	ShengliF1	SLF1
Chunmanqinggengcai	CMQGC	Shensizisong	SSZS
Gaojiaobaicai	GJBC	Wenzhoubaiyoudong	WZBYD
Ganxuanheiyebaicai	GXBC	Ziseqingcai102	ZSQC102
HuoqingcaiF1	HQCF1	Zaoshu5	ZS5
Hangzhouyoudonger	HZYDE	Zhenqing60F1	ZQ60F1
Meiguanqinggengcai	MGQGC	Zhouyewuyoudonger	ZYWYDE

**Table 2:** Analysis of variance for shoot biomass, Cd concentration, As concentration, Cd bioaccessibility in gastric phase, Cd bioaccessibility in gastrointestinal phase, As bioaccessibility in gastric phase and As bioaccessibility in gastrointestinal phase, of 20 genotypes under different levels of Cd and As co-contaminated soils ^a^.

Source of Variation	df	Shoot Biomass	Cd Concentration	As Concentration	Cd Bioaccessibility in Gastric Phase
Genotypes (G)	19	151.61 ^b^	114.77 ^b^	35.792 ^b^	53.56 ^b^
Soil treatments (S)	1	752.97 ^b^	3693.83 ^b^	788.76 ^b^	533.39 ^b^
G × S	19	20.63 ^b^	95.26 ^b^	20.65 ^b^	15.65 ^b^
		Cd bioaccessibility in gastrointestinal phase	As bioaccessibility in gastric phase	As bioaccessibility in gastrointestinal phase	
Genotypes (G)	19	78.26 ^b^	25.01 ^b^	44.94 ^b^	
Soil treatments (S)	1	994.35 ^b^	550.02 ^b^	908.47 ^b^	
G × S	19	29.50 ^b^	46.72 ^b^	38.82 ^b^	

^a^ Data presented are F-test values. ^b^ Significant at the 0.05 and 0.01 probability levels.

**Table 3:** The bioaccessible value of estimated daily intake (BEDI) (μg kg^−1^ bw·day^−1^) for Cd and As from consumption of pakchoi cultured on low-level co-contaminated soil (L-C soil), and high-level co-contaminated soil (H-C soil).

Genotypes	BEDI of Cd in Vegetables				BEDI of As in Vegetables			
	L-C Soil		H-C Soil		L-C Soil		H-C Soil	
	Adult	Children	Adult	Children	Adult	Children	Adult	Children
MGQGC	0.09	0.11	0.31	0.37	0.05	0.06	0.14	0.16
ZQ60F1	0.18	0.22	0.34	0.41	0.04	0.05	0.14	0.16
GXBC	0.12	0.15	0.41	0.49	0.07	0.09	0.54	0.65
SLF1	0.24	0.29	0.50	0.60	0.04	0.05	0.22	0.26
SJQBC	0.26	0.31	0.62	0.74	0.10	0.12	0.17	0.21
ZSQC102	0.17	0.20	0.66	0.79	0.09	0.11	0.19	0.23
SSZS	0.33	0.40	0.66	0.79	0.05	0.06	0.15	0.18
ZYWYDE	0.09	0.11	0.67	0.81	0.11	0.13	0.17	0.20
CHQGC	0.63	0.76	0.73	0.88	0.06	0.08	0.22	0.27
GJBC	0.35	0.42	0.74	0.89	0.15	0.18	0.32	0.39
HZYDE	0.15	0.18	0.83	0.99	0.08	0.10	0.21	0.25
WZBYD	0.15	0.18	1.05	1.27	0.07	0.08	0.35	0.42
ZS5	0.16	0.20	1.06	1.28	0.11	0.14	0.27	0.32
SKBC	0.38	0.45	1.08	1.30	0.08	0.10	0.23	0.27
CMQGC	0.38	0.45	1.18	1.42	0.04	0.05	0.26	0.32
MHF1	0.43	0.52	1.18	1.42	0.12	0.14	0.14	0.17
BJQSZQ	0.43	0.52	1.24	1.49	0.06	0.07	0.15	0.18
CGQGC	0.55	0.66	1.54	1.85	0.06	0.08	0.31	0.38
CGBC	0.70	0.84	0.89	1.08	0.08	0.09	0.23	0.27
HQCF1	0.77	0.93	2.11	2.54	0.06	0.07	0.15	0.18
Safe Value	0.83 ^a^				3 ^b^			

^a^ The previously established provisional tolerable monthly intake (PTMI) was 25 μg kg^−1^ bw (equivalent to 0.83 μg kg^−1^ bw day^−1^) according to the FAO/WHO [50]; ^b^ The provisional tolerable weekly intake (PTWI) of 21 μg kg^−1^ bw (equivalent to 3 μg kg^−1^ bw day^−1^) according to the FAO/WHO [50].
